# Association of Rare Copy Number Variants With Risk of Depression

**DOI:** 10.1001/jamapsychiatry.2019.0566

**Published:** 2019-04-17

**Authors:** Kimberley Marie Kendall, Elliott Rees, Matthew Bracher-Smith, Sophie Legge, Lucy Riglin, Stanley Zammit, Michael Conlon O’Donovan, Michael John Owen, Ian Jones, George Kirov, James Tynan Rhys Walters

**Affiliations:** 1 MRC Centre for Neuropsychiatric Genetics and Genomics, Division of Psychological Medicine and Clinical Neurosciences, Cardiff University, Cardiff, Wales, United Kingdom; 2 Centre for Academic Mental Health, Department of Population Health Sciences, University of Bristol, Bristol, United Kingdom

## Abstract

**Importance:**

The role of large, rare copy number variants (CNVs) in neuropsychiatric disorders is well established, but their association with common psychiatric disorders, such as depression, remains unclear.

**Objective:**

To examine the association of a group of 53 CNVs associated with neurodevelopmental disorders and burden of rare CNVs with risk of depression.

**Design, Setting, and Participants:**

This case-control study used data from the UK Biobank study sample, which comprised 502 534 individuals living in the United Kingdom. Individuals with autism spectrum disorder, intellectual disability, attention-deficit/hyperactivity disorder, schizophrenia, or bipolar affective disorder diagnoses were excluded. Analyses were further restricted to individuals of European genetic ancestry (n = 407 074). The study was conducted from January 2017 to September 2018.

**Exposures:**

CNV carrier status.

**Main Outcomes and Measures:**

For the primary outcome, individuals who reported that a physician had told them they had a depression diagnosis were defined as cases. Analyses were repeated using 2 alternative depression definitions: self-reported lifetime depression with current antidepressant prescription at the time of visit 1, and hospital discharge diagnosis of depression.

**Results:**

Copy number variants were identified in 488 366 individuals aged 37 to 73 years. In total, 407 074 individuals with European genetic ancestry (220 201 female [54.1%]; mean [SD] age of 56.9 [8.0] years) were included in the study. Of these individuals, 23 979 (5.9%) had self-reported lifetime depression and 383 095 (94.1%) reported no lifetime depression. The group of 53 neurodevelopmental CNVs was associated with self-reported depression (odds ratio [OR], 1.34; 95% CI, 1.19-1.49, uncorrected *P* = 1.38 × 10^−7^), and these results were consistent when using 2 alternative definitions of depression. This association was partially explained by physical health, educational attainment, social deprivation, smoking status, and alcohol consumption. A strong independent association remained between the neurodevelopmental CNVs and depression in analyses that incorporated these other measures (OR, 1.26; 95% CI, 1.11-1.43; *P* = 2.87 × 10^−4^). Eight individual CNVs were nominally associated with risk of depression, and 3 of these 8 CNVs (1q21.1 duplication, Prader-Willi syndrome duplication, and 16p11.2 duplication) survived Bonferroni correction for the 53 CNVs tested. After the exclusion of carriers of neurodevelopmental CNVs, no association was found between measures of CNV burden and depression.

**Conclusions and Relevance:**

Neurodevelopmental CNVs appear to be associated with depression, extending the spectrum of clinical phenotypes that are associated with CNV carrier status.

## Key Points

**Question:**

Are rare copy number variants associated with depression in a large population sample?

**Findings:**

In this case-control study of 407 074 individuals in the UK Biobank study, neurodevelopmental disorder copy number variants appear to be associated with the risk of depression in those without neurodevelopmental disorders. Physical health, educational attainment, social deprivation, smoking status, and alcohol consumption are variables that partially explain this association, and no evidence was found of an association between measures of copy number variant burden and depression.

**Meaning:**

Neurodevelopmental copy number variants appear to be associated with increases in the risk of depression in those without neurodevelopmental disorders.

## Teaser

This case-control study examines the variables that affect depression among individuals of European ancestry in the UK Biobank study who carry copy number variants associated with neurodevelopmental disorders.

## Introduction

The role of large rare copy number variants (CNVs) in neurodevelopmental disorders, including autism spectrum disorder, intellectual disability, attention-deficit/hyperactivity disorder, and schizophrenia, is well established.^1,2,3,4,5^ In schizophrenia, associations have been reported with both individual CNVs and an increased burden of rare deletions and duplications.^6,7,8^ In contrast, the association between CNVs and risk of depression remains unclear. Studies of CNVs in depression have been based on relatively small samples^9,10,11,12,13,14,15^ and have generated inconsistent results, and their findings have not met the criteria for genome-wide significance. The UK Biobank database now offers an opportunity to investigate the association between CNVs and depression in a well-phenotyped sample and on a larger scale than has been possible.

We report a CNV analysis of depression in the entire UK Biobank sample. Previous studies have shown that a substantial proportion of CNV enrichment in schizophrenia is explained by CNVs associated with neurodevelopmental disorders.^6,16^ Depression shares a genetic risk with schizophrenia^17,18^ and is a frequent comorbidity with neurodevelopmental disorders.^19,20^ Together, these findings suggest the hypothesis that, if CNVs play a role in depression, neurodevelopmental CNVs are those most likely to be associated. We also tested a more general hypothesis that, at a genome-wide level, CNV burden is associated with depression.

## Methods

This current study was conducted from January 2017 to September 2018, under the conditions of the UK Biobank project number 14421. Ethical approval was granted to UK Biobank by the North West Multi-Centre Ethics Committee, and all participants provided informed consent to participate in UK Biobank projects.

### Sample

Between 2006 and 2010, the UK Biobank study recruited 502 534 individuals (54% female) aged 37 to 73 years living in the United Kingdom. Phenotypic data were collected at assessment centers through touchscreen devices and nurse-led interviews. Participants provided blood, urine, and saliva samples.

### Depression Phenotypes

Analyses of depression in the UK Biobank to date have used multiple definitions of the disorder.^21,22^ In view of this lack of consensus on case definition, we used a relatively liberal definition of lifetime depression, rating as cases those individuals who reported that a physician had told them they have depression. We repeated our analyses using 2 alternative, more conservative definitions of depression: (1) self-reported lifetime depression with current antidepressant prescription at the time of visit 1 and (2) hospital discharge diagnosis of depression.

For self-reported depression with antidepressant prescription at visit 1, we constructed a binary depression variable using the self-reported depression code 1286 in UK Biobank field 20002 and the antidepressant prescription codes in UK Biobank field 20003. Individuals were included as cases (affected status) if they reported that a physician had told them they have depression and that they received a prescription for an antidepressant medication at the time of first assessment. Individuals who fulfilled only 1 of the 2 criteria (ie, self-reported depression or antidepressant prescription alone) were excluded from analyses.

**Figure 1.**
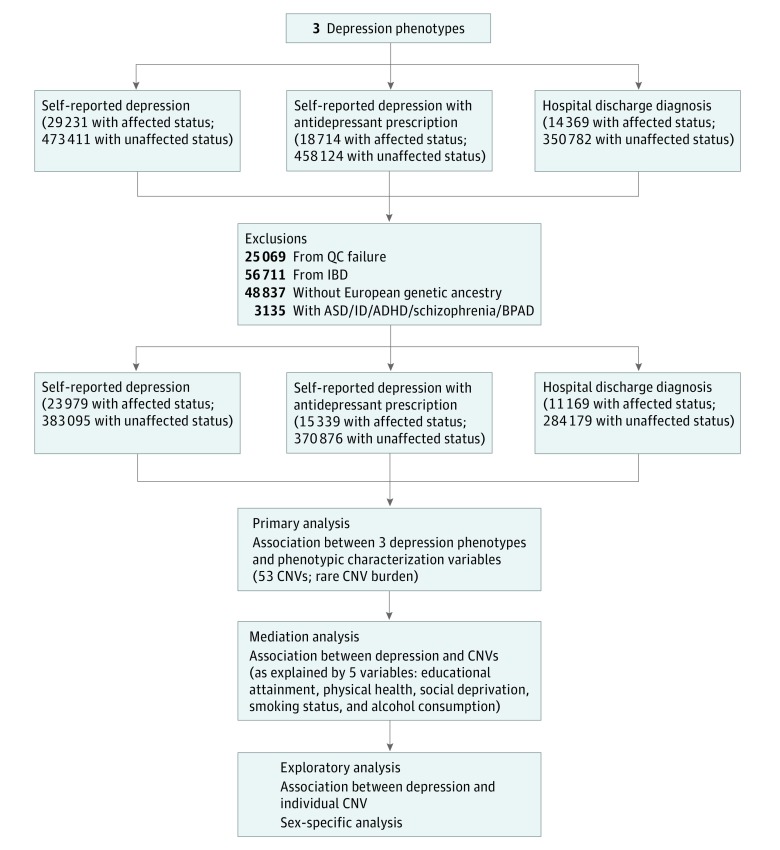
Flowchart of Study Methods ASD indicates autism spectrum disorder; ADHD, attention-deficit/hyperactivity disorder; BPAD, bipolar affective disorder; CNV, copy number variant; IBD, identity by descent; ID, intellectual disability; QC, quality control.

For hospital discharge diagnosis of depression, we included individuals as affected if they had a hospital admission with a primary or secondary *International Statistical Classification of Diseases and Related Health Problems, Tenth Revision (ICD-10)*, code for depression (UK Biobank fields 41202 and 41204) (Figure 1). For individuals assessed at Scottish assessment centers, records from general hospitals but not psychiatric hospitals were included (for assessment centers in Wales and England, the records covered both general and psychiatric hospitals). For these individuals, we accepted a secondary *ICD-10* code for depression as evidence of depression diagnosis as it could be coded during admission to a general hospital. However, in the absence of primary *ICD-10* codes for depression, we were unable to determine the absence of depression in controls (those with unaffected status). Therefore, we removed Scottish controls from this variable.

In 2017, a total of 157 397 individuals completed an online follow-up mental health questionnaire. We aimed to further characterize the associations with depression phenotypes in individuals who stated on this questionnaire that they had ever experienced prolonged feelings of sadness or depression (UK Biobank field 20446). We examined data from the following variables: (1) age at first episode of depression (UK Biobank field 20433), (2) duration of worst depression (UK Biobank field 20438), and (3) lifetime number of depressed episodes (UK Biobank field 20442). The duration of worst depression was coded in ranges of months (eg, less than a month, between 1 month and 3 months). Previous data have shown that the median duration of a depressive episode is 3 months.^23^ Therefore, this variable was dichotomized into 0 to 3 months and more than 3 months. The lifetime number of depressed episodes was dichotomized using a median split approach (median = 1).

### Genotyping and CNV Calling

DNA was extracted from whole blood^24^ and then genotyped (at the Affymetrix Research Services Laboratory) on the UK Biobank Axiom and UK BiLEVE arrays. Genotypes were released to Cardiff University after application to UK Biobank. We carried out CNV calling with PennCNV-Affy 1.0.3^25^ protocols (PennCNV) using biallelic markers common to both genotyping platforms; this process is described in detail elsewhere (eTable 6 in the Supplement).^26^ As reported in our group’s previous CNV analyses in UK Biobank, no batch effects were observed.^27^ The CNV burden analysis was carried out on the CNV calls generated by PennCNV-Affy using the PLINK 1.07 analysis toolset (eTable 7 in the Supplement).^28^ Individual samples were excluded if they had 30 or more CNVs, a waviness factor greater than 0.03 or less than –0.03, a single-nucleotide polymorphism call rate lower than 96%, or log R ratio SD higher than 0.35. Individual CNVs were excluded if they were covered by fewer than 20 probes, had a density coverage of less than 1 probe per 20 000 base pairs, or a confidence score lower than 10. eTable 9 in the Supplement provides the criteria for calling individual CNVs.

### Defining CNV Sets and Statistical Analysis

Following the approach of our group’s recent study using UK Biobank data,^26^ we defined a group of neurodevelopmental CNVs as those 54 CNVs for which at least a nominally statistically significant evidence of association with neurodevelopmental disorders exists (*P* < .05; eTable 11 in the Supplement).^3^ We excluded the high-frequency 15q11.2 duplication, resulting in a final list of 53 neurodevelopmental CNVs. The level of significance was set at *P* < .05. In exploratory analyses, we examined each of the CNVs with 5 or more observations for association with self-reported depression, and the results were subjected to correction for 53 tests (*P* value threshold of .00094).

The CNV burden analyses were carried out using PLINK on regions of variable copy number at 3 size thresholds: (1) 100 kilobase (kb) or greater, (2) 500 kb or greater, and (3) 1 megabase (Mb) or greater. The CNVs were filtered for frequency at less than 1% using the cnv-freq-exclude-above command, and the overlapping lower copy repeat regions were filtered out using the cnv-exclude command. PLINK outputs were converted into CNV carrier status, which was used in regression analyses. For all burden analyses, carriers of the group of 53 CNVs associated with neurodevelopmental disorders were excluded.

Association analyses were carried out in R (R Foundation for Statistical Computing) using logistic or linear regression as appropriate and with age, sex, genotyping array, and the first 15 principal components as covariates (eTable 8 in the Supplement). Analyses were restricted to individuals of European genetic ancestry (n = 407 074). This restriction was determined by calculating a minimum covariance determinant estimator of location and scatter, and then selecting individuals within the 90th percentile of the minimum covariance determinant distance (covMCD function in robustbase in R).^29,30^ We excluded individuals who had autism spectrum disorder, intellectual disability, attention-deficit/hyperactivity disorder, schizophrenia, or bipolar affective disorder diagnosis by a physician (UK Biobank fields 20002 and 20544) or diagnosis code during a hospital admission (UK Biobank fields 41202 and 41204).

### Further Investigation of the Neurodevelopmental CNV and Depression Association

To better understand the association between neurodevelopmental CNVs and depression, we investigated whether the association was explained by variables known to be associated with depression^31,32,33,34^ and postulated to be associated with CNVs: (1) educational attainment (qualifications), (2) physical health (affected status for 1 of the medical phenotypes associated with these CNVs),^27^ (3) social deprivation (Townsend deprivation index), (4) smoking (smoking status), and (5) alcohol consumption (alcohol intake frequency).

Prior to this analysis, data from the academic qualifications field were dichotomized and recoded into college or university degree or all other qualifications, an approach previously used for this data field (UK Biobank field 6138).^35^ For the physical health variable, we used affected status for the medical phenotypes associated with the CNVs in a recent work from our group.^27^ The social deprivation variable was measured using the Townsend deprivation index codes (UK Biobank field 189). Smoking was examined using smoking status (UK Biobank field 20116), and alcohol consumption was examined using alcohol intake frequency (UK Biobank field 1558).

Analyses were carried out using structural equation modeling in the lavaan package in R, which generates estimates of direct and indirect effects.^36^ The proportion explained was estimated by indirect effect divided by total effect.

### Sex-Specific Analyses

Previous studies have reported a small but significant excess of large (≥500-kb) rare (frequency of <1%) CNVs in female individuals.^37^ Recent evidence has also suggested that female children with anxiety or depression are more likely to carry large CNVs than male children.^38^ This finding led us to examine the rates of depression in female and male carriers of CNVs in our sample. With the finding that an excess of female carriers have depression, we added to the main regression model an interaction term consisting of the product of neurodevelopmental CNVs and sex.

## Results

We generated CNV calls for 488 366 individuals aged 37 to 73 years. In total, 407 074 individuals with European genetic ancestry (220 201 female [54.1%]; mean [SD] age of 56.9 [8.0] years) were included in the study. Of these individuals, 23 979 (5.9%) had self-reported lifetime depression and 383 095 (94.1%) reported no lifetime depression (Figure 1).

### Association Between CNVs and Depression

The group of 53 neurodevelopmental CNVs was associated with depression in the primary analysis (odds ratio [OR], 1.34; 95% CI, 1.19-1.49, uncorrected *P* = 1.38 × 10^−7^; Table). Of the individuals with depression, 363 (1.5%) carried at least 1 of the 53 neurodevelopmental CNVs compared with 4368 (1.1%) of controls. Analysis of the alternative depression phenotypes produced consistent results, with the effect size increasing with more conservative definitions of depression (Table). The association between the neurodevelopmental CNVs and self-reported depression remained after removing cases with a hospital discharge diagnosis of depression (OR, 1.35; 95% CI, 1.21-1.51; uncorrected *P* = 5.48 × 10^−8^). Restricting these analyses to the subset of 12 schizophrenia-associated CNVs generated similar results.^5,6^

**Table.** Association Analyses for Neurodevelopmental CNV and CNV Burden With 3 Depression Phenotypes in 407 074 Individuals^a^

CNV Type	Carrier, No. (%)	Self-reported Depression (n = 23 979)		Self-reported Depression With Antidepressant Prescription on Visit 1 (n = 15 339)		Hospital Discharge Diagnosis of Depression (n = 11 169 )	
		OR (95% CI)	Uncorrected *P* Value	OR (95% CI)	Uncorrected *P* Value	OR (95% CI)	Uncorrected *P* Value
Neurodevelopmental CNVs	4731 (1.2)	1.34 (1.19-1.49)	1.38 × 10^−7^	1.42 (1.25-1.62)	1.18 × 10^−7^	1.51 (1.30-1.75)	2.95 × 10^−8^
Carrier status of rare CNVs^b^							
≥100 kb	197 779 (48.6)	1.01 (0.98-1.03)	.58	1.02 (0.98-1.05)	.53	1.04 (1.00-1.08)	.04
≥500 kb	35 351 (8.9)	1.05 (1.005-1.10)	.03	1.08 (1.02-1.14)	.01	1.08 (1.01-1.15)	.03
≥1 Mb	13 946 (3.4)	1.01 (0.94-1.08)	.80	1.02 (0.94-1.12)	.59	1.04 (0.94-1.15)	.41

Abbreviations: CNV, copy number variant; kb, kilobase; Mb, megabase; OR, odds ratio.

^a^ Analyses were restricted to those of European genetic ancestry (n = 407 074) and excluded individuals with CNV-associated neurodevelopmental or neuropsychiatric disorders. Carriers of the group of 53 neurodevelopmental CNVs were excluded from CNV burden analyses.

^b^ Frequency less than 1%.

After the exclusion of carriers of the 53 neurodevelopmental CNVs, a weak association was found between CNVs of 500 kb or greater and depression, which did not survive correction for multiple testing. No evidence of an association between CNVs of 100 kb or greater and 1 Mb or greater and depression was found (Table).

**Figure 2.**
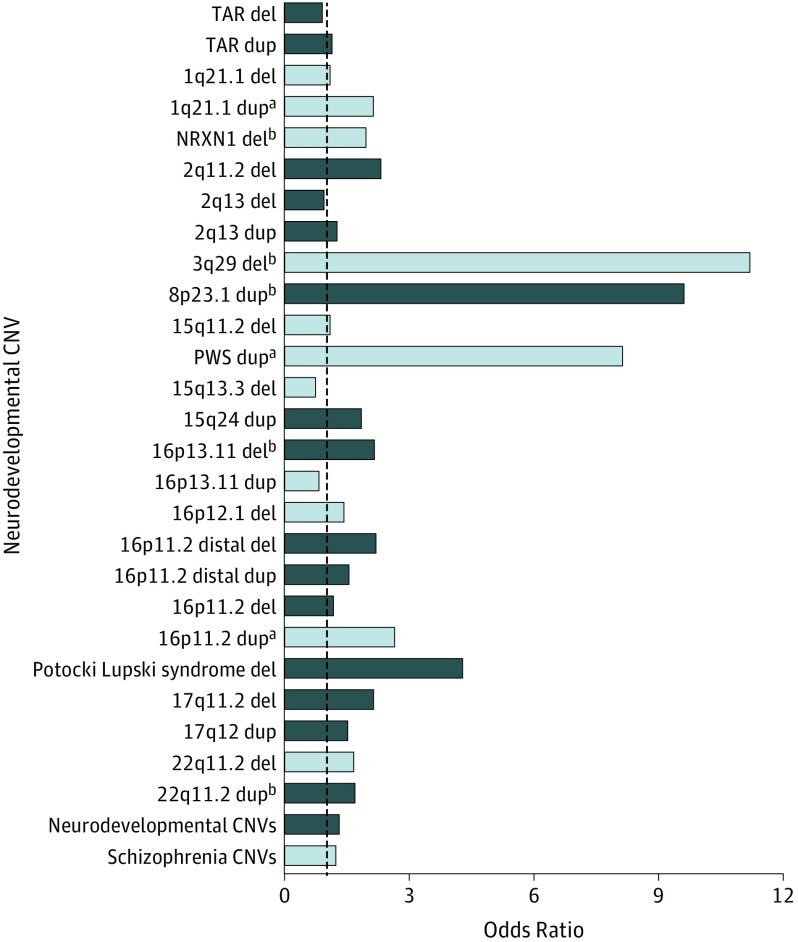
Analyses of Individual Neurodevelopmental Copy Number Variants (CNVs) for Association With Self-reported Depression Del indicates deletion; dup, duplication. ^a^CNVs that survived Bonferroni correction for 53 tests. ^b^Results with *P* < .05. Schizophrenia CNVs are shown as light blue. All CNVs fall within the neurodevelopmental CNV group. The dashed line indicates an odds ratio of 1.

Exploratory analysis of individual neurodevelopmental CNVs found 8 CNVs to be nominally associated with self-reported depression, of which 3 (1q21.1 duplication, Prader-Willi syndrome duplication, and 16p11.2 duplication) survived Bonferroni correction for the 53 neurodevelopmental CNVs tested (*P* value threshold of .00094; Figure 2 and eTable 1 in the Supplement).

### Association Between CNVs and Depression Severity

We used data from the 157 397 individuals who completed an online follow-up mental health questionnaire to examine the association between CNVs and markers of depression severity (age at onset, number of depressive episodes, and duration of worst depressive episode). We restricted these analyses to the 68 684 affected individuals who reported experiencing prolonged feelings of sadness or depression (57 243 unaffected), a phenotype that itself was associated with neurodevelopmental CNV carrier status (OR, 1.20; 95% CI, 1.07-1.36; *P* = .002). We did not find any association between neurodevelopmental CNVs and markers of depression severity that survived correction for multiple testing (eTable 2 in the Supplement). The mental health questionnaire provides potential alternative depression variables. Among these variables are a depression phenotype constructed using the Composite International Diagnostic Interview—Short Form, as reported by Davis et al,^39^ and an alternative self-reported depression variable. We examined these depression phenotypes for their association with neurodevelopmental CNVs and found consistent results when compared with the primary depression definitions, although the effect sizes were somewhat smaller, as would be expected for milder depression definitions (eTable 10 in the Supplement).

### Further Investigation of the Neurodevelopmental CNV and Depression Association

**Figure 3.**
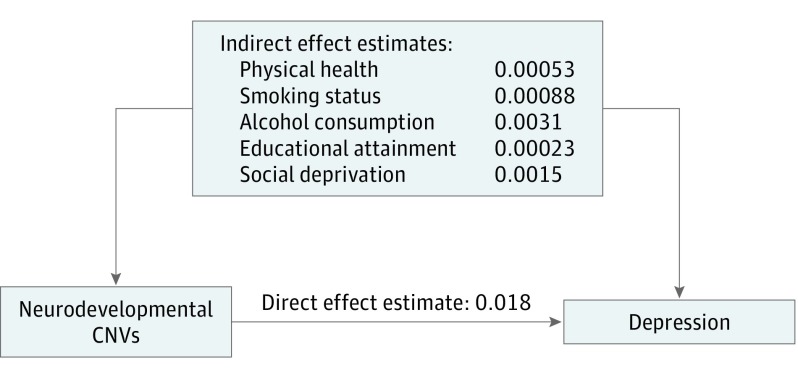
Association Between Neurodevelopmental Copy Number Variants (CNVs) and Depression The numbers shown are estimates for direct and indirect effects of each of the 5 variables (educational attainment, physical health, social deprivation, smoking status, and alcohol consumption), calculated using structural equation modeling in the lavaan package in R (R Foundation for Statistical Computing).^36^ Full results, including SEs and *P* values, are shown in eTable 3 in the Supplement.

To better understand the association between neurodevelopmental CNVs and depression, we investigated whether the association could be explained by measures of (1) educational attainment (qualifications), (2) physical health (presence or absence of an associated medical phenotype), (3) social deprivation (Townsend deprivation index), (4) smoking (smoking status), and (5) alcohol consumption (alcohol intake frequency). These variables were chosen because of their known associations with depression,^31,32,33,34^ their postulated or proven association with CNVs, and their availability in a large proportion of the UK Biobank sample. The association between neurodevelopmental CNVs and depression was partially explained by each variable examined: 1.2% explained by educational attainment; 2.9%, physical health; 8.1%, social deprivation; 4.8%, smoking status; and 16.6%, alcohol consumption (Figure 3 and eTable 3 in the Supplement). A strong independent association remained between the neurodevelopmental CNVs and depression in analyses that incorporated these other measures (OR, 1.26; 95% CI, 1.11-1.43; *P* = 2.87 × 10^−4^).

### Sex-Specific Analyses

Given the recent evidence of an increased rate of large CNVs in female children with anxiety or depression,^38^ we undertook an exploratory analysis, which provided weak evidence of a higher rate of depression among female carriers of neurodevelopmental CNV than among male carriers. This increased rate was over and above the baseline-increased rate of self-reported depression in females (interaction term OR, 0.66; 95% CI, 0.53-0.83; uncorrected *P* = .002; eTables 4 and 5 in the Supplement). However, this association was weaker for the secondary depression definitions.

## Discussion

To our knowledge, this research is the largest study of CNVs in depression to date. We performed a CNV analysis of depression in the UK Biobank sample of 407 074 individuals with European genetic ancestry. The primary definition of depression was an individual’s self-report of ever having received a medical diagnosis of depression (self-reported depression). To ensure that the findings were not restricted to this definition, we also tested 2 more conservative phenotypes: (1) lifetime self-reported depression with current antidepressant prescription at the time of visit 1 and (2) hospital discharge diagnosis of depression. We sought association with depression for a group of 53 CNVs known to be associated with neurodevelopmental disorders,^3^ and after excluding individuals with CNVs relevant to the primary hypothesis, we tested for a residual explained burden among CNVs of 100 kb or greater, 500 kb or greater, and 1 Mb or greater. The results support our first hypothesis that CNVs that were previously associated with neurodevelopmental disorders are associated with increased risk of lifetime depression, whether defined on the basis of self-reported diagnosis, self-reported diagnosis combined with antidepressant treatment, or on hospital discharge diagnosis. This analysis excluded those with a neurodevelopmental or neuropsychiatric diagnosis, and thus the association is unlikely to have been explained by associations with these disorders.

Three neurodevelopmental CNVs (1q21.1 duplication, Prader-Willi syndrome duplication, and 16p11.2 duplication) were individually associated with depression at levels of statistical significance, surviving Bonferroni correction for the 53 neurodevelopmental CNVs tested. None of these CNV loci overlap with risk loci recently identified in a large depression genome-wide association study.^18^ The risk of depression in CNV carriers in the current study (whether CNVs were considered individually or collectively) was lower compared with the risk identified in previous studies of schizophrenia. However, qualitatively, the results followed a similar pattern: The highest risk for both disorders was conferred by 3q29del (depression OR, 11.22 vs schizophrenia OR, 57.65) and the lowest risk for both disorders was conferred by 16p12.1del (depression OR, 1.47 vs schizophrenia OR, 3.3).^5,6^ After excluding neurodevelopmental CNVs, we found no evidence of residual burden of risk for depression among CNVs of 100 kb or greater, 500 kb or greater, and 1 Mb or greater.

Further investigation of the association between neurodevelopmental CNVs and depression risk indicated that this association is partially explained by educational attainment, social deprivation, physical health, smoking status, and alcohol consumption. To our knowledge, this is the first study to show these CNVs are associated with neighborhood measures of social deprivation, and thus it implicates an important mechanism by which CNV carrier status could increase the risk of depression. Longitudinal data on these measures are needed to establish the causal directionality between depression and social deprivation.

### Limitations

This study has some limitations. The primary depression definition relied on self-report, a method known to be subject to information bias.^40^ However, this definition is unlikely to have markedly affected the findings, given the almost identical, or even stronger, results from using the clinicians’ hospital discharge diagnosis of depression phenotype. Another limitation is the relatively low rate of depression compared with the population estimates.^41^ This lower rate may have been associated with the better-than-average health and functioning of the UK Biobank sample and the imprecise definition of depression. However, these factors were not likely to have generated spurious CNV associations and would instead have diluted the associations with CNV carrier status.

## Conclusions

Neurodevelopmental CNVs have incomplete penetrance for major developmental disorders,^42^ yet beyond their association with mild cognitive impairment,^26,43^ little is known about their phenotypic associations with CNV carrier status. This study, to our knowledge, is the first to robustly demonstrate the association between these CNVs and risk of depression and thus extends the spectrum of clinical phenotypes that are associated with CNV carrier status. This work reiterates that carriers of CNVs without neurodevelopmental disorders cannot be assumed to be unimpaired. It appears that along with cognitive and physical health manifestations, wider implications for depression and social deprivation must be considered in assessing CNVs at the population level.

## Notes

Supplement. **eTable 1**. Association Analyses of Individual Neurodevelopmental CNVs With Self-Reported Depression **eTable 2**. Analyses of Depression Sub-phenotypes for Individuals Who Stated They Had Ever Experienced Prolonged Feelings of Sadness or Depression **eTable 3**. Direct and Indirect Effect Results of Potential Explanatory Variables for the Neurodevelopmental CNV-Depression Association **eTable 4**. The Rates of Depression in Females and Males in (i) the Whole Sample, (ii) Carriers of Neurodevelopmental CNVs **eTable 5**. Association Analyses of Neurodevelopmental With the Three Depression Phenotypes According to Sex **eTable 6**. Commands and Parameters Used for the Processing of Genotype Data With Affymetrix Power Tools and PennCNV-Affy **eTable 7**. Examples of the Plink Code Used **eTable 8**. Examples of the R Code Used **eTable 9**. Criteria Used for Calling CNVs **eTable 10**. A Comparison of the Phenotypic Definitions Used in This Study and the Results of Association Analyses With Neurodevelopmental CNVs **eTable 11.** CNV Coordinates
