# Supplementary material for: Association of Rare Copy Number Variants With Risk of Depression
Source: JAMA Psychiatry. 2019 Apr 17;76(8):818–25. doi: 10.1001/jamapsychiatry.2019.0566 (PMC6583866; doi:10.1001/jamapsychiatry.2019.0566)
Supplement: Supplement. — eTable 1. Association Analyses of Individual Neurodevelopmental CNVs With Self-Reported Depression eTable 2. Analyses of Depression Sub-phenotypes for Individuals Who Stated They Had Ever Experienced Prolonged Feelings of Sadness or Depression eTable 3. Direct and Indirect Effect Results of Potential Explanatory Variables for the Neurodevelopmental CNV-Depression Association eTable 4. The Rates of Depression in Females and Males in (i) the Whole Sample, (ii) Carriers of Neurodevelopmental CNVs eTable 5. Association Analyses of Neurodevelopmental With the Three Depression Phenotypes According to Sex eTable 6. Commands and Parameters Used for the Processing of Genotype Data With Affymetrix Power Tools and PennCNV-Affy eTable 7. Examples of the Plink Code Used eTable 8. Examples of the R Code Used eTable 9. Criteria Used for Calling CNVs eTable 10. A Comparison of the Phenotypic Definitions Used in This Study and the Results of Association Analyses With Neurodevelopmental CNVs eTable 11. CNV Coordinates [file jamapsychiatry-76-818-s001.pdf]

Supplementary Online Content

Kendall KM, Rees E, Bracher-Smith M, et al. Association of rare copy number variants with risk of depression. *JAMA Psychiatry.* Published online April 17, 2019. doi:10.1001/jamapsychiatry.2019.0566

eTable 1. Association Analyses of Individual Neurodevelopmental CNVs With Self-Reported Depression

eTable 2. Analyses of Depression Sub-phenotypes for Individuals Who Stated They Had Ever Experienced Prolonged Feelings of Sadness or Depression

eTable 3. Direct and Indirect Effect Results of Potential Explanatory Variables for the Neurodevelopmental CNV-Depression Association

eTable 4. The Rates of Depression in Females and Males in (i) the Whole Sample, (ii) Carriers of Neurodevelopmental CNVs

eTable 5. Association Analyses of Neurodevelopmental With the Three Depression Phenotypes According to Sex

eTable 6. Commands and Parameters Used for the Processing of Genotype Data With Affymetrix Power Tools and PennCNV-Affy

eTable 7. Examples of the Plink Code Used

eTable 8. Examples of the R Code Used

eTable 9. Criteria Used for Calling CNVs

eTable 10. A Comparison of the Phenotypic Definitions Used in This Study and the Results of Association Analyses With Neurodevelopmental CNVs

eTable 11. CNV Coordinates

This supplementary material has been provided by the authors to give readers additional information about their work.

© 2019 Kendall KM et al. *JAMA Psychiatry*.

CNV	N	OR (95% CI)	p	CNV	N	OR (95% CI)	p
TAR del	70	0.95 (0.29 – 2.29)	0.92	15q24 dup	8	1.89 (0.10 – 10.74)	0.55
TAR dup	405	1.17 (0.77 – 1.69)	0.44	16p13.11 del	126	2.21 (1.25 – 3.63)	0.003
1q21.1 del	104	1.11 (0.46 – 2.22)	0.79	16p13.11 dup	791	0.87 (0.63 – 1.78)	0.39
1q21.1 dup	173	2.17 (1.34 – 3.36)	9.08×10^{-4} *	16p12.1 del	236	1.47 (0.90 – 2.27)	0.09
NRXN1 del	160	2.01 (1.18 – 3.19)	0.0057	16p11.2 distal del	57	2.23 (0.92 – 4.63)	0.05
2q11.2 del	31	2.34 (0.69 – 6.02)	0.11	16p11.2 distal dup	131	1.57 (0.82 – 2.73)	0.14
2q13 del	51	0.98 (0.24 – 2.67)	0.97	16p11.2 del	110	1.21 (0.54 – 2.34)	0.60
2q13 dup	68	1.29 (0.49 – 2.9)	0.59	16p11.2 dup	124	2.65 (1.53 – 4.31)	2.04×10^{-4} *
3q29 del	8	11.22 (2.27 – 46.52)	0.001	PL syndrome dup	5	4.31 (0.22 – 29.82)	0.19
8p23.1 dup	6	9.64 (1.32 – 50.21)	0.009	17q11.2 del	9	2.16 (0.12 – 11.86)	0.47
15q11.2 del	1578	1.11 (0.90 – 1.35)	0.30	17q12 dup	95	1.55 (0.69 – 3.02)	0.23
Prader Willi syn dup	17	8.14 (2.77 – 21.69)	4.61×10^{-5} *	22q11.2 del	10	1.69 (0.09 – 9.17)	0.62
15q13.3 del	43	0.77 (0.13 – 2.52)	0.72	22q11.2 dup	267	1.72 (1.12 – 2.53)	0.009

eTable 1. Association Analyses of Individual Neurodevelopmental CNVs With Self-Reported Depression

© 2019 Kendall KM et al. *JAMA Psychiatry*.

Analyses were restricted to those of genetic European ancestry and individuals with CNV-associated neurodevelopmental/neuropsychiatric disorders were excluded. Logistic regression analyses included age, sex, genotyping array and the first 15 principal components as covariates. N – number of CNV carriers (affected and unaffected combined), OR – odds ratio, 95% CI – 95% confidence interval, p – uncorrected p value. Results, which survived Bonferroni correction for 53 tests are marked with *. Some CNVs were not analysed because they (i) were not observed 1p36 dup, Wolf-Hirschhorn del, Wolf-Hirschhorn dup, Soto syndrome del, 9q34 dup (*EHMT1*), Potocki-Shaffer del, 15q24 del, 17p13.3 del (*YWHAE*), 17p13.3 dup (*YWHAE*), 17p13.3 del (*PAFAH1B1*), 17p13.3 dup (*PAFAH1B1*), 17q21.31 del, *SHANK3* del, *SHANK3* dup) (ii) had counts < 5 (1p36 del, 2q37 del, Williams Beuren del, 8p23.1 del, 10q23 del, Prader Willi syn del, 15q25 del, Smith Magenis syn del, 17q11.2 dup (*NF1*), 22q11.2 distal del), or (iii) no carriers had depression (Williams Beuren dup, 17q12 del, 22q11.2 distal del).

© 2019 Kendall KM et al. *JAMA Psychiatry*.

Variable	CNV Class	Effect Size (95% CI)	p
20446 Ever had prolonged feelings of sadness or depression (68,684 affected, 57,243 unaffected)	Neurodevelopmental CNVs	1.20 (1.07 – 1.36)	0.002
	100KB + <1%	0.99 (0.98 – 1.02)	0.96
	500KB+ <1%	0.99 (0.95 – 1.03)	0.65
	1MB + <1%	0.96 (0.90 – 1.02)	0.25
20433 Age at first episode of depression (n =65,106)	Neurodevelopmental CNVs	-0.07 (-0.15 - -0.002)	0.06
	100KB + <1%	-0.001 (-0.016 – 0.014)	0.86
	500KB + <1%	0.002 (-0.027 – 0.030)	0.91
	1MB + <1%	-0.005 (-0.047 – 0.037)	0.82
20438 Duration of worst depression (0-3 months vs > 3 months) (n =69,971)	Neurodevelopmental CNVs	0.98 (0.84 – 1.14)	0.74
	100KB + <1%	0.99 (0.97 – 1.03)	0.78
	500KB + <1%	0.99 (0.94 – 1.05)	0.82
	1MB + <1%	1.07 (0.98 – 1.17)	0.10
20442 Lifetime number of depressed episodes (1 vs 2+) (n =57,482)	Neurodevelopmental CNVs	1.04 (0.87 – 1.24)	0.68
	100KB + <1%	1.00 (0.97 – 1.04)	0.86
	500KB + <1%	1.02 (0.96 – 1.09)	0.51
	1MB + <1%	1.13 (1.03 – 1.25)	0.009

© 2019 Kendall KM et al. *JAMA Psychiatry*.

eTable 2. Analyses of Depression Sub-phenotypes for Individuals Who Stated They Had Ever Experienced Prolonged Feelings of Sadness or Depression (UKBB field 20446)

Analyses used logistic regression with age, sex, genotyping array and the first 15 principal components as covariates except that for age at first episode of depression (UKBB field 20433), which used linear regression (same covariates). Analyses were restricted to those of genetic European ancestry, and individuals with CNV-associated neurodevelopmental/neuropsychiatric disorders were excluded from being cases and controls. Effect size – odds ratio (except for age at first episode of depression – standardised Beta), 95% CI – 95% confidence interval, p – uncorrected p value.

© 2019 Kendall KM et al. *JAMA Psychiatry*.

**Indirect Effects**	Proportion Explained	Estimate	Standard Error	p
**Explanatory Variable**				
Educational attainment	1.2%	0.00023	7.78×10^{-5}	0.0032
Physical health	2.9%	0.00053	1.85×10^{-4}	0.0045
Social deprivation	8.1%	0.0015	1.59×10^{-4}	< 0.0001
Smoking	4.8%	0.00088	1.58×10^{-4}	0.000000029
Alcohol consumption	16.6%	0.0031	2.39×10^{-4}	< 0.0001
**Sum of Indirect Effects**	21.5%	0.0039	3.85×10^{-4}	< 0.0001
**Direct Effect**				
Neurodevelopmental CNVs		0.018	0.0038	0.0000025

eTable 3. Direct and Indirect Effect Results of Potential Explanatory Variables for the Neurodevelopmental CNV-Depression Association

Direct and indirect effect results of analyses aiming to examine whether the association between neurodevelopmental CNVs and depression could be explained by educational attainment, physical health, social deprivation, smoking status or alcohol consumption. All results were generated using structural equation modelling in Lavaan. The proportion explained was estimated by indirect effect/total effect. SE – standard error, p – uncorrected p value.

© 2019 Kendall KM et al. *JAMA Psychiatry*.

	Females	Males	Chi square	p
Whole Sample				
Rate of depression	7.16% (n = 15,761)	4.39% (n = 8,218)	1388.97	3.37×10^{-304}
CNV Carriers Only				
Rate of depression	10.24% (n = 246)	5.02% (n = 117)	45.45	1.22×10^{-11}

eTable 4. The Rates of Depression in Females and Males in (i) the Whole Sample, (ii) Carriers of Neurodevelopmental CNVs

Analyses were restricted to those of genetic European ancestry, and individuals with CNV-associated neurodevelopmental/neuropsychiatric disorders were excluded from being cases and controls. CNV carriers are included in the 'whole sample' section of the table. p – p value.

© 2019 Kendall KM et al. *JAMA Psychiatry*.

CNV Type	Self-Reported Depression<br><br>23,979 affected<br><br>383,095 unaffected		Self-Reported Depression and Antidepressant Prescription on Visit 1<br>15,339 affected,<br><br>370,876 unaffected		Hospital Discharge Diagnosis of Depression<br>11,169 affected<br><br>284,179 unaffected	
	OR (95% CI)	p	OR (95% CI)	p	OR (95% CI)	p
Neurodevelopmental CNVs						
All (females and males)	1.34<br><br>(1.19 – 1.49)	1.38×10^{-7}	1.42<br><br>(1.25 – 1.62)	1.18×10^{-7}	1.51<br><br>(1.30 – 1.75)	2.95×10^{-8}
Females	1.46<br>(1.28 – 1.67)	2.23×10^{-8}	1.51<br>(1.29 – 1.78)	3.75×10^{-7}	1.67<br>(1.39 – 2.01)	2.66×10^{-8}
Males	1.14<br>(0.95 – 1.38)	0.17	1.27<br>(1.01 – 1.59)	0.041	1.28<br>(0.99 – 1.63)	0.052
Interaction term (product of neurodevelopmental CNVs and sex)	0.66<br>(0.53 – 0.83)	0.0002	0.67<br>(0.51 – 0.87)	0.003	0.67<br>(0.49 – 0.91)	0.009

eTable 5. Association Analyses of Neurodevelopmental With the Three Depression Phenotypes According to Sex

Analyses were restricted to those of European genetic ancestry and excluded individuals with CNV-associated neurodevelopmental/neuropsychiatric disorders. OR – odds ratio, 95% CI – 95% confidence interval, p – uncorrected p value.

© 2019 Kendall KM et al. *JAMA Psychiatry*.

Command	Parameters
apt-probeset-genotype	/apt-probeset-genotype --analysis-files-path /Axiom UKB WCSG.r3/ --xml-file /Axiom UKB WCSG 96orMore Step2 Bi-allelic.r3.apt-probeset-genotype.AxiomGT1.xml --out-dir /Batch1 --summaries --cel-files cel.list batch1.txt
generate affy geno cluster.pl	/generate affy geno cluster.pl AxiomGT1.calls.txt AxiomGT1.confidences.txt AxiomGT1.summary.txt --nopower2 -locfile mapfile.dat -sexfile sex batch1.txt -out batch1.genocluster
normalize affy geno cluster.pl	normalize affy geno cluster.pl batch1.genocluster AxiomGT1.summary.txt -nopower2 -locfile mapfileAX.dat -out batch1 lrr baf.txt
kcolumn.pl	kcolumn.pl batch1 lrr baf.txt split 2 -start 1 -end 1000 -tab -head 3 -name
ls	ls split1*>Batch1 signalfilelist
compile pfb.pl	compile pfb.pl -listfile Batch10 500signalfilelist.txt -output
detect cnv.pl	detect cnv.pl -test -hmm Axiom trained.hmm –pfb Axiom.pfb -listfile Batch1 signalfilelist -out --confidence --log –gcmodel Axiom.gcmodel
clean cnv.pl	clean cnv.pl combineseg Batch1.rawcnv > join batch1.rawcnv -signalfile Axiom.pfb –fraction 0.25 -bp

eTable 6. Commands and Parameters Used for the Processing of Genotype Data With Affymetrix Power Tools and PennCNV-Affy (given arbitrarily for batch 1)

© 2019 Kendall KM et al. *JAMA Psychiatry*.

CNV Type	Code
Rare CNVs 500kb (deletions and duplications)	./plink --cnv-list burden.txt --map burden.map --fam srd.ped.txt --cnv-kb 500 --cnv-freq-exclude-above 4070 --cnv-exclude exclregions.txt --cnv-overlap 0.5 --cnv-indiv-perm --mperm 10000
Rare CNVs 500kb (deletions)	./plink --cnv-list burden.txt --map burden.map --fam srd.ped.txt --cnv-kb 500 --cnv-del --cnv-freq-exclude-above 4070 --cnv-exclude exclregions.txt --cnv-overlap 0.5 --cnv-indiv-perm --mperm 10000
Rare CNVs 500kb (deletions)	./plink --cnv-list burden.txt --map burden.map --fam srd.ped.txt --cnv-kb 500 --cnv-dup --cnv-freq-exclude-above 4070 --cnv-exclude exclregions.txt --cnv-overlap 0.5 --cnv-indiv-perm --mperm 10000

eTable 7. Examples of the Plink Code Used

(given arbitrarily for 500kb CNVs and the self-reported depression phenotype)

© 2019 Kendall KM et al. *JAMA Psychiatry*.

	Code
Logistic regression model	`model <- glm(srd ~ ndcnv + age + sex + array + pc1 + pc2 + pc3 + pc4 + pc5 + pc6 + pc7 + pc8 + pc9 + pc10 + pc11 + pc12 + pc13 + pc14 + pc15, family=binomial(link='logit'), data=depression)`
Generate odds ratio	`exp(coefficients(model))`
Generate 95% confidence intervals for the odds ratio	`exp(cbind(OR= coef(model), confint(model)))`
Lavaan model	model <- '#outcome model<br>dep ~ c*ndcnv + b1*degree + b2*tdi + b3*dx + b4*alcohol + b5*smoking<br><br>#mediator models<br>degree ~ a1*ndcnv<br>tdi ~ a2*ndcnv<br>dx ~ a3*ndcnv<br>alcohol ~ a4*ndcnv<br>smoking ~ a5*ndcnv<br><br>#indirect effects<br>degreeIDE := a1*b1<br>tdiIDE := a2*b2<br>dxIDE := a3*b3<br>alcoholIDE := a4*b4<br>smokingIDE := a5*b5<br>sumIDE := (a1*b1) + (a2*b2) + (a3*b3) + (a4*b4) + (a5*b5)

© 2019 Kendall KM et al. *JAMA Psychiatry*.

	#total effect<br>total := c + (a1*b1) + (a2*b2) + (a3*b3) + (a4*b4) + (a5*b5)'
Model fitting	fit <- sem(model, data=mediation)
Parameter estimates	boot.fit <- parameterEstimates(fit, boot.ci.type = "bca.simple")

eTable 8. Examples of the R Code Used

Lavaan code requires the installation of the Lavaan package.

© 2019 Kendall KM et al. *JAMA Psychiatry*.

CNV	Criteria
1p36 del/dup	Size >50% of critical region, affecting *GABRD*
TAR del/dup	Size >50% of critical region
1q21.1 del/dup	Size >50% of critical region
NRXN1 del	Exonic deletions
2q11.2 del/dup	Size >50% of critical region, affecting both *LMAN2L* and *ARID5A*
2q13 del/dup	Size >50% of critical region
2q13 del/dup (*NPHP1*)	Size >50% of critical region, affecting *NPHP1*
2q21.1 del/dup	Size >50% of critical region
2q37 del/dup (*HDAC4*)	Size >50% of critical region, affecting *HDAC4*
3q29 del/dup	Size >50% of critical region
Wolf-Hirschhorn del/dup	Size >50% of critical region
Sotos Syn/5q35 dup	Size >50% of critical region
6q16 del/dup (*SIM1*)	Exonic deletions; whole gene duplications
Williams Beuren Syn del/dup	Size >50% of critical region
7q11.23 distal del/distal dup	Size >50% of critical region
8p23.1 del/dup	At least 1Mbp of critical region
9q34 del/dup (*EHMT1*)	At least 1Mbp CNVs, including *EHMT1*
10q11.21q11.23 del/dup	Size >50% of critical region
10q23 del/dup	At least 1Mbp, including *NRG3* and *GRID1*
Potocki-Shaffer Syn del/11p11.2 dup (*EXT2*)	Size >50% of critical region, including *EXT2*
13q12 del/dup (*CRYL1*)	Exonic deletions; whole gene duplications
13q12.12 del/dup	Size >50% of critical region
15q11.2 del/dup	Size >50% of critical region
PWS del/dup	Full critical region, ~4Mbp
15q11q13 del/dup BP3-BP4	Size >50% of critical region
15q11q13 del/dup BP3-BP5	Size >50% of critical region
15q13.3 del/dup	Size >50% of critical region

© 2019 Kendall KM et al. *JAMA Psychiatry*.

15q13.3 del/dup (*CHRNA7*)	Size >50% of critical region, affecting *CHRNA7*
15q24 del/dup	At least 1Mbp between the A-E intervals
15q25 del/dup	At least 1Mbp between the A-D intervals
Rubinstein-Taybi del/dup (*CREBBP*)	Exonic deletions; whole gene duplications
16p13.11 del/dup	Size >50% of critical region
16p12.1 del/dup	Size >50% of critical region
16p12.2-p11.2 del/dup (7.1-8.7 Mb)	Size >50% of critical region
16p11.2 distal del/distal dup	Size >50% of critical region
16p11.2 del/dup	Size >50% of critical region
17p13.3 del/dup (*YWHAE*)	Exonic deletions; whole gene duplications
17p13.3 del/dup (*PAFAH1B1*)	Exonic deletions; whole gene duplications
17p12 del (HNPP)/dup (CMT1A)	Size >50% of critical region, affecting *PMP22*
Smith-Magenis/Potocki-Lupski Syndrome	Size >50% of critical region
17q11.2 del/dup (*NF1*)	Size >50% of critical region, affecting *NF1*
17q12 del/dup	Size >50% of critical region
17q21.31 del/dup	Size >50% of critical region
17q23.1q23.2 del/dup	Size >50% of critical region
22q11.2 del/dup	Size >50% of critical region
22q11.2 distal del/dup	Size >50% of critical region
SHANK3 del/dup	At least 1Mbp CNVs, including *SHANK3*

eTable 9. Criteria Used for Calling CNVs

© 2019 Kendall KM et al. *JAMA Psychiatry*.

Phenotype	Criteria	OR (95% CI)	p
Self-reported depression (assessment centre)	Self-report of a doctor diagnosis of depression – code 1286 in field 20002.<br>Exclusions – BPAD, SCZ, ASD, ID, ADHD.	1.34 (1.19 – 1.49)	1.38×10^{-7}
Self-reported depression and antidepressant prescription at visit 1	Self-report of a doctor diagnosis of depression – code 1286 in field 20002 and antidepressant prescription at the time of assessment (field 20003).<br>Exclusions - BPAD, SCZ, ASD, ID, ADHD, individuals fulfilling one criterion (i.e. depression alone or antidepressant prescription alone).	1.42 (1.25 – 1.62)	1.18×10^{-7}
Hospital discharge diagnosis of depression	Hospital admission with a primary or secondary ICD-10 code for depression (fields 41202 and 41204).<br>Exclusions – BPAD, SCZ, ASD, ID, ADHD.	1.51 (1.30 – 1.75)	2.95×10^{-8}
Self-reported depression diagnosis (MHQ)[1]	Self-report of a depression diagnosis from a health professional.<br>Exclusions – none.	1.29 (1.03 – 1.62)	0.025
Constructed CIDI depression variable (MHQ)[1]	Persistent sadness 20446 **or** loss of interest field 20441 **and**,<br>most of day or all day long 20436 **and**,<br>felt this way almost every day or every day 20439 **and**,<br>somewhat impaired or a lot of impairment 20440 **and,**<br>total number of symptoms ≥ 5 - persistent sadness 20446, loss of interest 20441, tired/low energy 20449, gain/loss/gain and loss of weight 20536, sleep change 20532, trouble concentrating 20435, feeling worthless 20450, thinking about death 20437<br>Exclusions – none.	1.13 (0.99 – 1.29)	0.064

© 2019 Kendall KM et al. *JAMA Psychiatry*.

eTable 10. A Comparison of the Phenotypic Definitions Used in This Study and the Results of Association Analyses With Neurodevelopmental CNVs

BPAD – bipolar affective disorder, SCZ – schizophrenia, ASD – autism spectrum disorder, ID – intellectual disability, ADHD – attention deficit hyperactivity disorder, OR – odds ratio, 95% CI – 95% confidence interval, p – uncorrected p value.

© 2019 Kendall KM et al. *JAMA Psychiatry*.

**Syndrome**	**Locus**	**Critical/Unique Sequence Region (hg19)**	**Coe_significant**	**Sz-CNV**
1p36 del (*GABRD*)	1p36	chr1:0-2500000	1	
1p36 dup (*GABRD*)	1p36	chr1:0-2500000	1	
TAR del	1q21.1	chr1:145,394,955-145,807,817	1	
TAR dup	1q21.1	chr1:145,394,955-145,807,817	1	
1q21.1 del	1q21.1	chr1:146,527,987-147,394,444	1	1
1q21.1 dup	1q21.1	chr1:146,527,987-147,394,444	1	1
NRXN1 del	2p16.3	chr2:50145643-51259674	1	1
2q11.2 del (*LMAN2L, ARID5A*)	2q11.2	chr2:96,742,409-97,677,516	1	
2q13 del	2q13	chr2:111,394,040-112,012,649	1	
2q13 dup	2q13	chr2:111,394,040-112,012,649	1	
2q37 del (*HDAC4*)	2q37	chr2:239,716,679-243,199,373	1	
3q29 del	3q29	chr3:195,720,167-197,354,826	1	1
Wolf-Hirschhorn del	4p16.3	chr4:1,552,030-2,091,303	1	
Wolf-Hirschhorn dup	4p16.3	chr4:1,552,030-2,091,303	1	
Sotos syndrome del	5q35	chr5:175,720,924-177,052,594	1	
Williams-Beuren syndrome (WBS) del	7q11.23	chr7:72,744,915-74,142,892	1	
WBS dup	7q11.23	chr7:72,744,915-74,142,892	1	1

© 2019 Kendall KM et al. *JAMA Psychiatry*.

8p23.1 del	8p23.1	chr8:8,098,990-11,872,558	1	
8p23.1 dup	8p23.1	chr8:8,098,990-11,872,558	1	
9q34 dup (*EHMT1*)	9q34	chr9:140,513,444-140,730,578	1	
10q23 del (*NRG3, GRID1*)	10q22q23	chr10:82,045,472-88,931,651	1	
Potocki-Shaffer syndrome del (*EXT2*)	11p11.2	chr11:43,940,000-46,020,000	1	
15q11.2 del BP1-BP2	15q11.2	chr15:22,805,313-23,094,530	1	1
15q11.2 dup BP1-BP2	15q11.2	chr15:22,805,313-23,094,530	1	
Prader-Willi syndrome/Angelman syndrome (PWS/AS) del	15q11.2q12	chr15:22,805,313-28390339	1	
PWS/AS dup	15q11.2q12	chr15:22,805,313-28390339	1	1
15q13.3 del BP4-BP5	15q13.3	chr15:31,080,645-32,462,776	1	1
15q24 del	15q24	chr15:72900171-78151253	1	
15q24 dup	15q24	chr15:72900171-78151253	1	
15q25 del	15q25.2	chr15:83,219,735-85,722,039	1	
16p13.11 del	16p13.11	chr16:15,511,655-16,293,689	1	
16p13.11 dup	16p13.11	chr16:15,511,655-16,293,689	1	1
16p12.1 del (520kb)	16p12.1	chr16:21,950,135-22,431,889	1	1
16p11.2 distal del (220kb)	16p11.2	chr16:28,823,196-29,046,783	1	
16p11.2 distal dup (220kb)	16p11.2	chr16:28,823,196-29,046,783	1	
16p11.2 del (593kb)	16p11.2	chr16:29,650,840-30,200,773	1	

© 2019 Kendall KM et al. *JAMA Psychiatry*.

16p11.2 dup (593kb)	16p11.2	chr16:29,650,840-30,200,773	1	1
17p13.3 del (*YWHAE*)	17p13.3	chr17:1,247,834-1,303,556	1	
17p13.3 dup (*YWHAE*)	17p13.3	chr17:1,247,834-1,303,556	1	
17p13.3 del (*PAFAH1B1*)	17p13.3	chr17:2,496,923-2,588,909	1	
17p13.3 dup (*PAFAH1B1*)	17p13.3	chr17:2,496,923-2,588,909	1	
Smith-Magenis syndrome del	17p11.2	chr17:16,812,771-20,211,017	1	
Potocki-Lupski syndrome dup	17p11.2	chr17:16,812,771-20,211,017	1	
17q11.2 del (*NF1*)	17q11.2	chr17:29,107,491-30,265,075	1	
17q11.2 dup (*NF1*)	17q11.2	chr17:29,107,491-30,265,075	1	
Renal cysts and diabetes syndrome del (RCAD)	17q12	chr17:34,815,904-36,217,432	1	
17q12 dup	17q12	chr17:34,815,904-36,217,432	1	
17q21.31 del	17q21.31	chr17:43,705,356-44,164,691	1	
22q11.2 del	22q11.2	chr22:19,037,332-21,466,726	1	1
22q11.2 dup	22q11.2	chr22:19,037,332-21,466,726	1	
22q11.2 distal del	22q11.2	chr22:21,920,127-23,653,646	1	
22q11.2 distal dup	22q11.2	chr22:21,920,127-23,653,646	1	
SHANK3 del	22q13	chr22:51113070-51171640	1	
SHANK3 dup	22q13	chr22:51113070-51171640	1	

eTable 11. CNV Coordinates

© 2019 Kendall KM et al. *JAMA Psychiatry*.
